# A case report of a rare intrahepatic cholangiocarcinoma with rectal metastasis

**DOI:** 10.3389/fonc.2025.1548570

**Published:** 2025-06-05

**Authors:** Shu-min Jiang, Lu Huang, Quan Jiang

**Affiliations:** Department of Hepatobiliary Surgery, The First Affiliated Hospital of Jinan University, Guangzhou, China

**Keywords:** intrahepatic cholangiocarcinoma, rectal metastasis, cancer treatment, tumor marker, pathological features

## Abstract

Intrahepatic cholangiocarcinoma (ICC) is a primary liver cancer with insidious onset, rapid progression, and poor prognosis. The lymphatic system is the main route of ICC distant metastasis, with lungs, adrenal glands, and brain as the most common extrahepatic sites. However, extrahepatic metastases of ICC have rarely been reported in patients with rectal symptoms as initial symptoms, and the diagnosis relies on the specific immunohistochemical features of intestinal lesion biopsy. Herein, this study presents an incidental case of ICC with rectal metastasis to investigate its characteristics based on its diagnosis, metastasis, and treatment.

## Introduction

Intrahepatic cholangiocarcinoma (ICC) is a rare primary liver malignancy. The significance of ICC lies in its rarity, poor patient prognosis, and high recurrence rate even with aggressive surgical treatment. The 5-year survival rate of ICC patients has been reported to be 13%–42%. The recurrence site of ICC generally includes the residual liver, lung, and peritoneum. There are also rare case reports of ICC metastasis to the gastrointestinal tract worldwide. Due to its low incidence, once most are found to be advanced, there are considerable challenges to the treatment and survival of doctors and patients. Here, we hope to provide the therapeutic experience of patients with ICC gastrointestinal metastasis through a profound analysis of the diagnosis and treatment of this rare case.

## Case presentation

**Figure 1:**
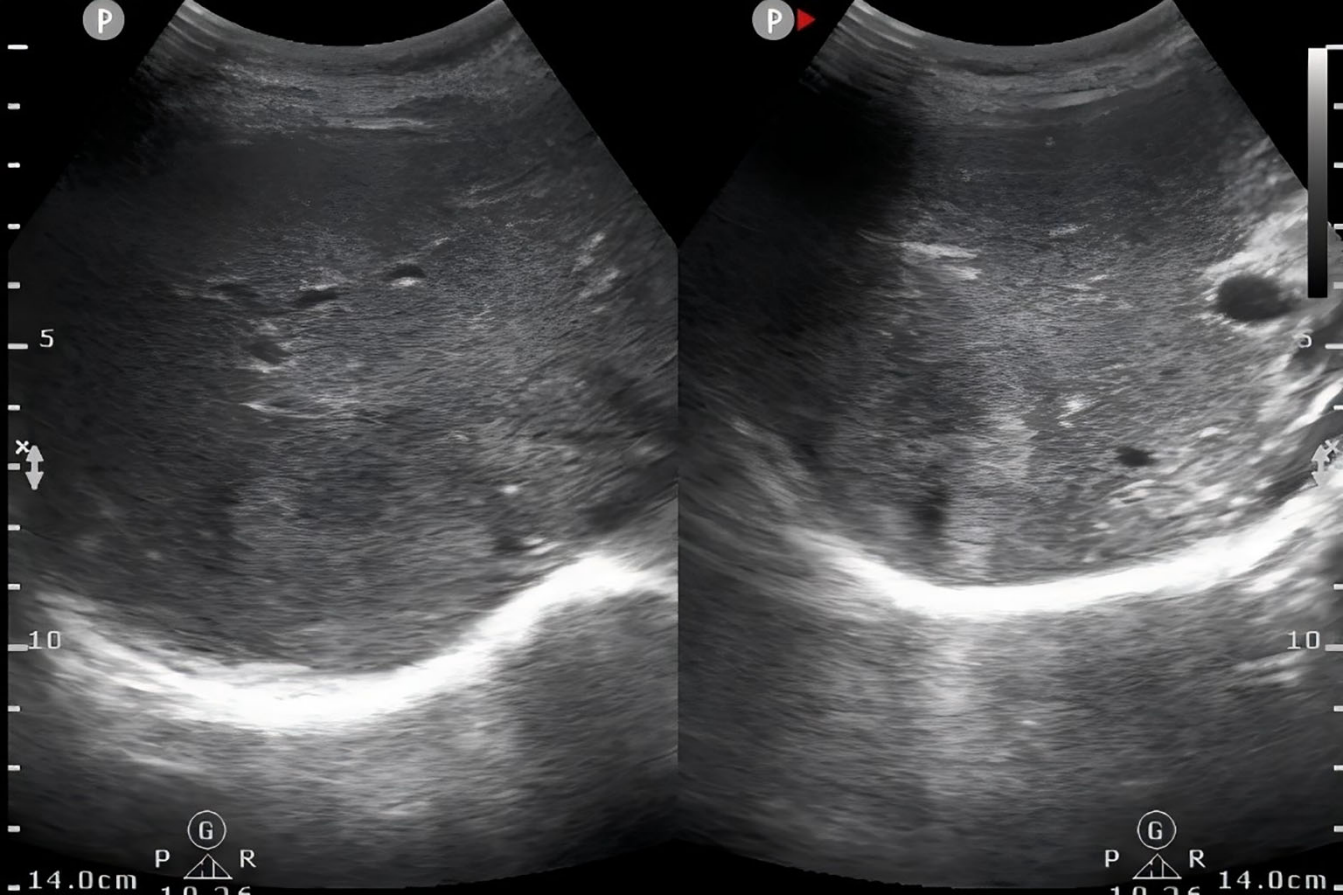
Abdominal ultrasonography showed heterogeneous hypoechoic nodules in right lobe of liver (S7,8).

**Figure 2:**
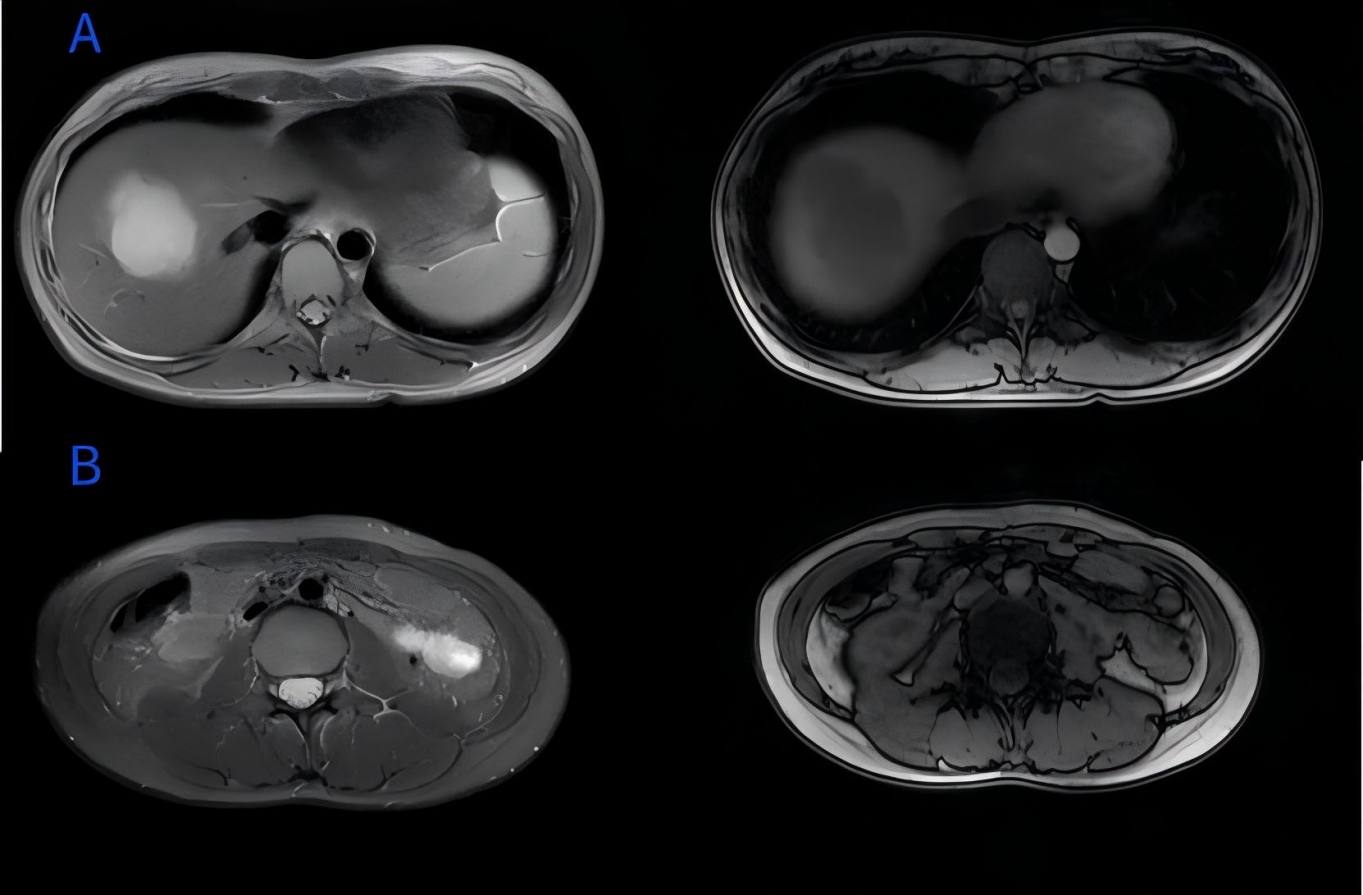
**(A)** Abdominal-enhanced magnetic resonance imaging (MRI) showed intrahepatic lesion with rim enhancement in S8 segment of liver; **(B)** abdominal MRI showed rectosigmoid mass with heterogeneous enhancement (red arrow).

A 28-year-old female was admitted to the hospital with altered bowel habits and stool forms for more than 1 month. Her investigations in another hospital with abdominal CT revealed wall thickening and soft tissue mass at the rectosigmoid junction and a mass shadow in the right lobe of the liver, suggesting liver metastasis of colon cancer in the right lobe; colonoscopy showed a mucosal bulge in the rectum; endoscopic ultrasound revealed bulging and narrowing of the rectum, suggesting compression by an extramural mass and the possibility of focal intestinal wall invasion; and tumor markers showed CA-125 of 490 IU/ml. She was primarily diagnosed with a malignant rectal tumor. She had a family history of liver cancer developed in her father, whereas other history was unremarkable. Physical examination did not reveal abnormal findings. Pertinent laboratory findings revealed that glutathione reductase (GR-K) was 88.33 U/L, α-hydroxybutyrate dehydrogenase (α-HBDH) was 238 U/L, lactate dehydrogenase (LDH) was 408 U/L, prealbumin (PA) was 136.0 mg/l, cancer antigen 125 (CA-125) was 560.8 U/ml, cancer antigen 15-3 (CA15-3) was 42.2 U/ml, carcinoembryonic antigen (CEA) was 1.02 ng/ml, and α-fetoprotein (AFP) was 4.73 ng/ml. Abdominal ultrasonography with color Doppler showed heterogeneous hypoechoic nodules in the right lobe (S7,8) of a normal-sized liver, suspecting a metastatic tumor, and a hypoechoic space-occupying lesion in the pelvic cavity (**Figure 1**). Plain and enhanced abdominal magnetic resonance imaging (MRI) revealed a rectosigmoid segment with irregular wall thickening and a mass of suspected T4 rectal cancer with an ill-defined border, adjacent small intestine involvement, local extramural growth, and heterogeneous enhancement, while an intrahepatic lesion was 6.3 cm × 4.6 cm in size with rim enhancement, suggesting a metastatic tumor (**Figure 2**).

**Figure 3:**
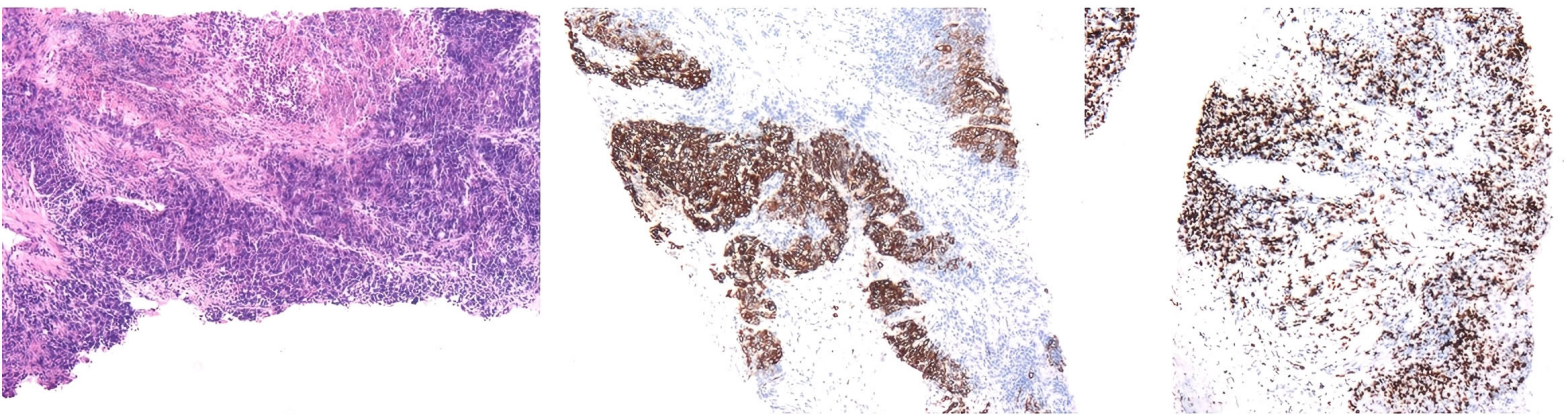
Pathological results of liver needle biopsy showed carcinoma infiltration of normal liver tissue. Immunohistochemical staining showed that CK7 (+), CK20 (−), CDX-2 (−), Syn (−), CgA (−), Ki-67 ~70% (+), PAX-8 (+), SATB 2 (−), P40 (individual, +), TTF-1 (−), WT-1 (−), P16 (partial, +), P53 (+) in about 80%.

The patient underwent ultrasound-guided needle biopsy of the liver mass on 21 March 2023. The pathological examination showed poorly differentiated carcinoma infiltration with necrosis and localized adenoid differentiation. Immunohistochemical stainings revealed consistency with a poorly differentiated adenocarcinoma and strongly suggested Müllerian duct origin, by showing CK7 (+), CK20 (−), CDX-2 (−), Syn (−), CgA (−), Ki-67 ~70% (+), PAX-8 (+), SATB 2 (−), P40 (individual, +), TTF-1 (−), WT-1 (−), P16 (partial, +), P53 (+) in about 80% (**Figure 3**).

After multidisciplinary team (MDT) discussion, conversion regimens were applied first with TACE and mFOLFOX6 chemotherapy prescribed for liver and intestinal tumors. On day 5 of discharge after completing the first chemotherapy course, this patient was admitted to the hospital again with mechanical intestinal obstruction resulting from rectosigmoid malignancy. As the patient showed a strong desire to preserve the anus, MDT discussion suggested an application of neoadjuvant radiotherapy before radical surgery. The applied regimen was 330 cGy per daily fraction, 5 days per week, delivered over 2 weeks with 1-day rest after five continuous treatments. After the second course of radiotherapy, the patient experienced aggravated abdominal pain and reduced defecation and flatulence. It was suspected that radiotherapy induced congestion and edema of the colon tumor, aggravating the obstruction. Hence, the radiotherapy was suspended.

**Figure 4:**
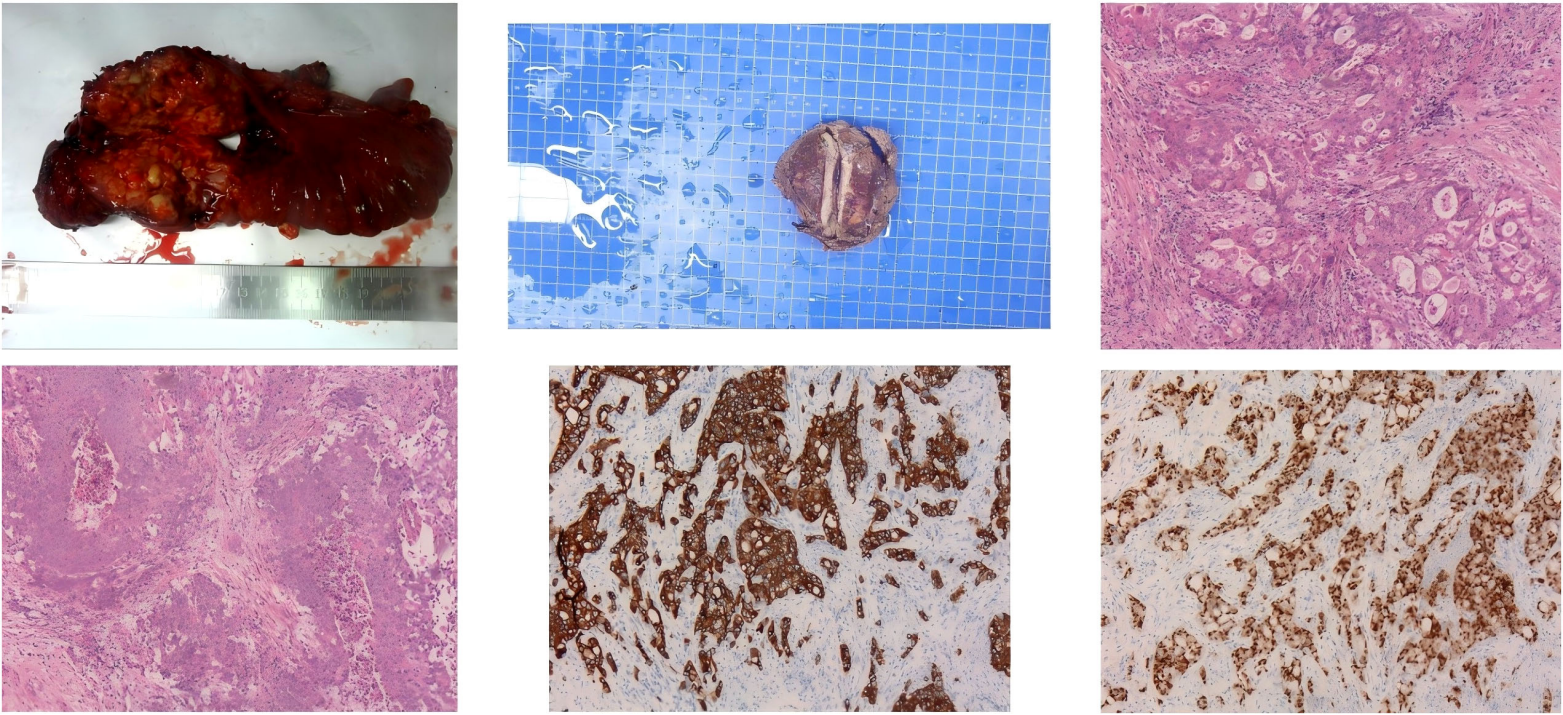
Intraoperative resection of tumor tissue and postoperative pathological results.

On 5 April 2023, the patient underwent laparoscopic radical resection of rectal cancer, ileostomy, liver mass resection, peritoneal adhesiolysis, and bilateral cystoscopic retrograde ureteric stent insertion. Postoperative pathological results showed an extramural rectal tumor with necrosis and significant desmoplasia, while IHC showed PAX-8 (+), PAX-2 (−), CK7 (+), CK20 (−), CK5/6 (−), CDX-2 (−), GATA-3 (−), Calretinin (−), D2-40 (−), WT-1 (−), GCDFP-15 (−), AR (−), ER (minimal, +), NapsinA (−), and Ki-67 (+) in about 30%, which was consistent with metastatic adenocarcinoma and liver tumor with diffuse necrosis (**Figure 4**). Based on the patient’s medical history and pathology, the diagnosis was confirmed as small duct ICC with rectal metastasis. The patient began an integrative therapy half a month after surgery by combining Oxaliplatin 100 mg with Gemcitabine 1400 mg, Sintilimab Injection 200 mg, and Anlotinib 8 mg once daily. Ileostomy reversal was performed during the third postoperative month, and no tumor recurrent metastasis was identified in the 14-month postoperative follow-up.

## Discussion

ICC, a rare epithelial malignancy originating from the intrahepatic bile duct, accounts for about 10%–15% of primary liver cancer (PLC) (1). ICC commonly metastasizes by lymphatic system, hematogenous spread, and peritoneal invasion, whereas seeding metastasis is the least common. Tumor cells invade the liver sinusoids, forming portal vein and hepatic vein tumor thrombi and leading to intrahepatic and extrahepatic metastasis, and the most common extrahepatic site is the lung. When liver cancer is complicated with extrahepatic metastasis, the most common initial complaint manifests with symptoms of liver injury, while extrahepatic metastases or colorectal cancers as initial complaints have rarely been reported. Only four related articles reporting simultaneous occurrence of ICC and colorectal tumors were found after a thorough review of current literature.

Metastatic liver disease is a malignancy originating from one part of the body and spreading to the liver, resulting in solitary or multiple liver lesions. The unique biliary ductal anatomy and abundant blood flow of the liver make it one of the most common metastatic sites of other primary malignancies, and liver metastases are more prominent than PLC. Among all malignancies, colorectal cancer is the most common origin of liver metastasis, followed by breast, pancreas, lung, stomach, and so on (2). When liver metastases are detected, most patients manifest without specific symptoms or present with symptoms of primary cancer only.

Identification of the primary lesion and site of origin is of paramount importance for subsequent treatment and prognosis in cancer patients. In this case, the most important task is to determine whether the tumor has a liver or colon origin. The patient presented with initial symptoms of intestinal obstruction at admission, and the abnormal liver enhancement in radiological examinations suggested the possibility of metastatic tumor. Combined with the epidemiological characteristics, rectal cancer with liver metastasis was highly suspicious. Literature suggested the differentiation between primary and metastatic rectal cancer by endoscopy, pathological examination, and immunohistochemical staining. Endoscopically, metastatic rectal cancer manifests as submucosal nodules with intact mucosa and luminal narrowing, thereby presenting with intestinal obstruction, which is consistent with this case (3), whereas primary rectal cancer manifests as focal lobulated lesions with hemorrhagic ulcers. Immunohistochemical staining with CA19–9 and CEA are the most commonly used serum markers for ICC diagnosis, which can be used for diagnosis and predicting prognosis. However, CA19–9 level is often affected by biliary obstruction, and suspicions of tumor diseases should be considered for continuous elevation of CA19–9 after excluding biliary obstruction (4). CK-7 and CK-20 are specific immunohistochemical indicators for differentiating primary and metastatic rectal cancer and a reliable indicator for differentiating ICC from primary intestinal-type adenocarcinoma (5). Research revealed positive CK-20 in 70%–95% of colorectal carcinomas and in 20%–40% of pancreaticobiliary adenocarcinomas and positive CK-7 in 90%–100% of pancreaticobiliary adenocarcinoma and in 5%–25% of colorectal adenocarcinomas (6). Meanwhile, CDX-2 is highly specific and sensitive in gastrointestinal adenocarcinoma, and a negative CDX-2 profile contributes to the exclusion of primary intestinal adenocarcinoma (7). The immunophenotype of the patient was highly consistent with CK7 (+), CK20 (−), and CDX-2 (−), confirming that the rectal adenocarcinoma was not primary but a metastatic ICC.

The metastatic rate of ICC is related to the tumor size, growth, and host immunity. Since the liver has an abundant vascular structure and peripheral lymph distribution, ICC is prone to intrahepatic and extrahepatic metastases, and distant metastasis occurs in 70% of clinical autopsies (8). Unlike HCC, which mainly metastasizes hematogenously via the portal vein (9), ICC metastasizes to abdominal lymph nodes by lymphatic reflux and extrahepatic organs by seeding. The primary lymphatic route of ICC metastasis begins from intrahepatic lymph nodes to hepatic hilar lymph nodes, followed by portacaval lymph nodes, celiac lymph nodes, and finally reaching abdominal paraaortic lymph nodes, which is often manifested as multiple skip lymph nodes metastasis. Among them, hepatic hilar lymph node metastasis is the most common. According to the latest NCCN guidelines, when unresectable features were identified during surgical exploration, such as lymph node metastasis beyond hepatic hilar lymph, distant metastasis, and local advanced tumor, then delayed surgical treatment is recommended (10). We performed lymph node dissection at the time of surgery. However, no metastases were identified in hepatic hilar and abdominal paraaortic lymph nodes, and only two metastatic lymph nodes could be detected surrounding the intestine, which is rarely seen clinically. Thus, skip metastasis of cancer cells through the lymphatic system to the rectum was highly suspected. Lymph node metastasis often suggests a poor prognosis (11). However, postoperative pathology suggested small duct type ICC, which had a better prognosis than large duct type, and this patient did not show any signs of tumor recurrence in 14-month postoperative follow-up. Therefore, despite the presence of lymph node metastasis, a good prognosis can yet result if the pathological classification tends to be small duct type ICC (12).

Radical surgical resection is currently the only possible curative method for ICC, with a 5-year survival rate of about 30% and a 5-year recurrence rate of 60%–70% (13). As treatment methods become more diverse, surgery-oriented integrative interventions have been formed, including surgical resection (radical and palliative resection), liver transplantation, ablation therapy (radiofrequency, microwave, absolute ethanol), interventional therapy, radiotherapy, molecular targeted/immunotherapy, chemotherapy, and other treatment methods. In this case, the rectal cavity was compressed and narrowed, resulting in difficult defecation and obvious symptoms. Considering the patient’s age and the pelvic mass location, surgical resection may remove the anus, and the postoperative quality of life would reduce significantly. Therefore, interventional embolization was performed at the beginning of treatment to embolize both liver and pelvic tumors. Surgical resection was then carried out after pelvic tumor shrinkage to preserve anal function. When neoadjuvant therapy showed unsatisfactory effects, radical surgical treatment was performed after considering the resectability of the rectal cancer lesion and liver metastatic lesion, preservation of sufficient liver tissue, and the patient’s tolerability. Gemcitabine- and platinum-based combination chemotherapy is recommended as the first-line treatment in ICC patients with positive findings in lymph node dissection (14). Gemcitabine-based combination chemotherapy can reduce the clinical stage of unresectable ICC, and the prognosis of subsequent radical resection is favorable (15). The rapid development of immunotherapy and targeted therapy in recent years provides a basis for the newly explored triple therapy regimen (immune checkpoint inhibitor + tyrosinase inhibitor + chemotherapy) to significantly improve tumor treatment efficacy and conversion rate (16). Postoperative adjuvant chemotherapy is essential for patients with stage IV or locally advanced, unresectable, recurrent, or metastatic rectal cancer. Recommended chemotherapy regimens include the oxaliplatin-based CapeOx or FOLFOX regimen or single-agent therapy with 5-FU/LV or capecitabine. Therefore, combination therapy with oxaliplatin, gemcitabine, cindilizumab, and anlotinib was applied postoperatively, and the treatment was effective according to the patient’s postoperative follow-up.

## Conclusions

ICC with rectal metastasis is a rare mode of distant metastasis that may pose a diagnostic challenge. Although diagnosis may be difficult, primary and metastatic rectal cancer must be differentiated when rectal tumors are identified, as this may affect surgical treatment, prognosis, and quality of life. Several endoscopic and pathological features that distinguish primary rectal cancer from metastatic rectal cancer identified in ICC are supported by this case report and relevant literature. Meanwhile, this case report presents the entire treatment process with a personalized treatment regimen that takes into account the patient’s systemic and local efficacy, as well as the short-term and long-term prognosis.

## Data Availability

The original contributions presented in the study are included in the article/supplementary material. Further inquiries can be directed to the corresponding author.
